# Interleukin-6: a potent biomarker of mycobacterial infection

**DOI:** 10.1186/2193-1801-2-686

**Published:** 2013-12-21

**Authors:** Prati Pal Singh, Amit Goyal

**Affiliations:** Centre of Infectious Diseases, Department of Pharmacology and Toxicology, National Institute of Pharmaceutical Education and Research, S. A. S. Nagar, 160 062 Punjab, India

**Keywords:** Biomarker, Cytokines, Interleukin-6, Macrophage, Mycobacteria

## Abstract

**Background:**

Human tuberculosis (TB), a chronic inflammatory disease is caused by *Mycobacterium tuberculosis*, a facultative intramacrophage pathogen. The highly complex interactions between mycobacteria and macrophages (MΦs), characterized in part by the induction and elaboration of several cytokines including IL-1, IL-6, IL-10, IL-12 p40 and IL-12 p70 are not yet fully understood. The cytokines are known to have important bearing on the pathogenesis and host defense during TB. We thus studied different patterns of cytokines elaborated by mouse peritoneal macrophages (PMs) following their interaction with live and heat-killed, virulent and avirulent, and pathogenic and non-pathogenic mycobacteria, *in vitro*.

**Materials and methods:**

Pathogenic *M. tuberculosis* H37Rv (virulent) and *M. tuberculosis* H37Ra (avirulent), and non-pathogenic *M. smegmatis* were grown in complete Middle Brook 7H9 broth. For some experiments, mycobacteria were heat-killed (80°C; 20 min). The supernatants of cultured PMs, having ingested mycobacteria for 6 h, 24 h, 4 days and 7 days, were harvested for the quantification of IL-1, IL-6, IL-10, IL-12 p40 and IL-12 p70 by using a multiplex suspension cytokine array system.

**Results:**

The PMs infected with heat-killed mycobacteria, as compared to their respective live counterparts, invariably elaborated significantly (p < 0.001) increased (approximately 2–3-fold) amounts of IL-6, at all the time-points studied, *in vitro*. Further, PMs infected with *M. tuberculosis* H37Ra, as compared to *M. tuberculosis* H37Rv, elaborated 4–5-fold more (p < 0.001) IL-6. Non-pathogenic *M. smegmatis*, as compared to pathogenic *M. tuberculosis* H37Ra and *M. tuberculosis* H37Rv, following infection, induced the PMs to elaborate highest (p < 0.001) amounts of IL-6 at all the time-points studied. Curiously, none of these mycobacteria-infected PMs elaborated IL-1, IL-10, IL-12 p40 and IL-12 p70, significantly.

**Conclusion:**

IL-6 appears to be the only major cytokine elaborated by mycobacteria-infected PMs, *in vitro*, and thus may function as a potent biomarker of mycobacterial infection, either stand-alone or along with other cytokines.

## Introduction

Globally, tuberculosis (TB) continues to be a major public health problem, and is responsible for an estimated 1.4 million deaths and 8.7 million new cases every year (World Health Organization, 2012). TB, caused by *Mycobacterium tuberculosis*, a slow-growing facultative pathogen, is a prototypical re-emerging infectious disease in the developed countries, whereas it is a major cause of morbidity and mortality in the developing countries (North and Jung, 2004). The world TB situation is continually turning from bad to worse due to the emergence of multidrug-resistant (MDR) and virtually untreatable extensively drug-resistant (XDR) strains of *M. tuberculosis* (World Health Organization, 2012; Shah et al., 2007). The emergence and unabated spread of human-immunodeficiency virus (HIV) among TB patients has added new formidable dimensions to the problem of TB. During TB, macrophages (MΦs) are the first host cells to interact with *M. tuberculosis*, and function as the major habitat center for its intracellular multiplication and growth. The interaction of MΦs, one of the key elements involved in immunity to TB, with various mycobacterial strains is known to ensue in the differential induction and elaboration of several pro-inflammatory [interleukin-1β (IL-1β), IL-6, IL-12, tumor necrosis factor-α (TNF-α), granulocyte-macrophage (GM) colony-stimulating factor (CSF; GM-CSF), granulocyte-CSF (G-CSF)] and anti-inflammatory cytokines including IL-10. The intricate interplay of these cytokines is thought to orchestrate the induction and progression of an effective innate anti-mycobacterial immune response.

Immunity and inflammation are intertwined processes. IL-1, IL-6, IL-12 p40 and IL-12 p70 are known to exert pro-inflammatory effects. IL-1 has been reported to protect hosts against various infections, and is involved in the onset and progression of an acute-phase response (Dinarello, 1996). Double-knockout (KO) mice, deficient in both IL-1α and IL-1β, following infection with *M. tuberculosis* H37Rv, have been reported to develop larger granulomatous lesions (Yamada et al., 2000), whereas IL-1 type 1 receptor knockout mice were highly susceptible, showed greater mycobacterial load, and developed granulomas with fewer MΦs and lymphocytes, and abundant granulocytes (Juffermans et al., 2000). IL-6 is a multifunctional cytokine with at least three major reported functions. First, IL-6, together with TNF-α and IL-1β, initiates early pro-inflammatory responses (Van Snick, 1990), and is a known inducer of acute-phase proteins (APPs) (Singh and Kaur, 2005). Second, IL-6 is involved in the promotion of T-cell and B-cell responses (Van Snick, 1990). Third, IL-6 participates in hematopoiesis (Van Snick, 1990). IL-6 has also been shown to play a role in the priming of a TB subunit vaccine (Leal et al., 2001). IL-6 deficient mice have been reported to be highly susceptible and ultimately succumbed to TB (Ladel et al., 1997). Additionally, IL-6 has been reported to be required for interferon-γ (IFN-γ)-induced protection against *M. tuberculosis* infection in mice (Saunders et al., 2000). Paradoxically, IL-6 is also known to promote the intracellular growth of *M. avium* (Shiratsuchi et al., 1991), and to inhibit the production of TNF-α and IL-1β (Schindler et al., 1990). IL-12, known to stimulate the growth of NK cells and T-cells, enhances the production of cytokines, and connects innate and adaptive immune responses against mycobacterial infections (Trinchieri, 1995). IL-12 has been reported to exert its protective effects in *M. tuberculosis*-infected mice, mainly through IFN-γ (Cooper et al., 1997). Therapeutic effect of transgenic tomato over-expressing IL-12 has been reported in a murine model of progressive pulmonary TB (Elías-López et al., 2008). IL-12 receptor deficiency has been reported to be a cause for lethality in disseminated MDR adult TB (Tabarsi et al., 2011). On the other hand, transgenic mice over-expressing IL-10 have been reported to be highly susceptible to *M. avium* infection, as compared to the Wild-type ones (Feng et al., 2002). IL-10 has also been thought to be responsible for the suppression of immune response against TB, and persistence of a long-term *M. tuberculosis* infection (Redford et al., 2011). IL-10 gene polymorphism plays a major role in susceptibility to or protection against TB (Ben-Selma et al., 2011). Increased anti-mycobacterial immunity has been observed in IL-10 deficient mice, which shows that IL-10 is an inhibitor of early mycobacterial clearance (Murray and Young, 1999). The present studies were undertaken to delineate the elaboration of IL-1β, IL-6, IL-10, IL-12 p40 and IL-12 p70 by mouse peritoneal macrophages (PMs) infected with *M. tuberculosis* H37Rv, *M. tuberculosis* H37Ra and *M. smegmatis*, *in vitro*, in terms of their potential to function as a biomarker(s). Our results, for the first time, demonstrate that IL-6 can be used as a biomarker of mycobacterial infection, either stand-alone or along with other cytokines.

## Materials and methods

### Mycobacterial cultures

*M. tuberculosis* H37Rv, *M. tuberculosis* H37Ra and *M. smegmatis* were grown in complete 7H9 broth (Hi-Media Laboratories Pvt. Ltd., Mumbai, India) supplemented with 1% glycerol (Hi-Media Laboratories), 0.05% Tween-80 (Sigma Aldrich, St. Louis, MO, USA) and 10% Middlebrook OADC enrichment (Difco, Sparks, MO, USA). The mycobacterial growth was monitored spectrophotometrically. For use in experiments, a single-cell bacterial suspension was prepared (by sonication at 20 KHz; 10 s; 10 cycles), gently agitated, and allowed to stand at room temperature for 10 min to allow the mycobacterial clumps to settle-down. The top part of the mycobacterial suspension was then adjusted to 1 McFarland standard, and diluted in antibiotic-free Dulbecco modified Eagle’s medium (DMEM; PAA laboratories GmbH, Austria) containing 2 mM L-glutamine (PAA laboratories GmbH), 0.01 M HEPES (Hi-Media laboratories) and 1 × 10^-4^ M 2-mercaptoethanol (Sigma Aldrich, St. Louis, MO, USA). To this, 1% heat-inactivated fetal bovine serum (FBS; PAA laboratories) was added. In some experiments, heat-killed (at 80°C in a water bath; 20 min) mycobacteria were used.

### Mice

Swiss mice (either sex; 18–20 g), obtained from the Central Animal Facility of the institute, were maintained at 22–24°C and 12 h light/dark cycle, with food and water provided *ad libitum*. All the experiments were done in accordance with the guidelines for the care and use of animals in scientific research, Indian National Science Academy, New Delhi, as adapted and promulgated by the Institutional Animal Ethics Committee.

### Macrophage culture

Mouse PMs were harvested as described (Kaur et al., 2004). Briefly, thioglycolate (4% wt/vol., 0.5 ml/mouse, 96 h; Sigma Aldrich)-elicited mouse peritoneal exudate cell suspension in chilled antibiotic-free DMEM was centrifuged (700 × *g*; 4°C; 7 min) and, the cell pellet was re-suspended (2 × 10^6^ cells/well) in the same medium supplemented with 10% FBS (CDMEM) and adjusted to 1 × 10^6^ cells/ml, and then 2 ml of the cell suspension was transferred to the wells of sterile 24-well tissue culture plates. The plates were incubated overnight at 37°C in a 5% CO_2_ incubator for cell adherence, and the non-adherent cells were washed-off with warm Hank’s balanced salt solution (HBSS). For T-cell removal, the adherent PMs were treated with rabbit anti-mouse T-cell serum (1:20; 4°C; 1 h), washed (×1) with warm HBSS and then incubated with rabbit complement (37°C; 1 h). As determined by LAL assay, the DMEM and HBSS contained < 0.1 ng/ml endotoxin. The PMs were > 96% pure according to morphologic, phagocytic and non-specific esterase staining criteria, and were > 98% viable as judged by trypan blue (Sigma Aldrich) exclusion.

### Infection of PMs

For infection, PM monolayers were exposed to both live and heat-killed *M. tuberculosis* H37Rv, *M. tuberculosis* H37Ra and *M. smegmatis* (multiplicity-of-infection, 1:10), separately, at 37°C for 4 h. Later, the extracellular mycobacteria were washed (×3)-off by warm HBSS, and 2 ml of fresh CDMEM was added to each well. The culture plates were then incubated at 37°C in a 5% CO_2_ incubator for 6 h, 24 h, 4 days and 7 days. After specific periods of incubation, the PM culture supernatants were harvested, filter (0.22 μ)-sterilized, and stored frozen (-70°C) until use.

### Cytokine quantification

A multiplex cytokine 5-plex kit (IL-1β, IL-6, IL-10, IL-12 p40 and IL-12 p70) was utilized according to the manufacturer’s instructions (Bio-Rad, Hercules, CA, USA). The calibration curves were generated from the recombinant cytokine standards with serial dilutions in serum standard diluent. High and low reference points were included to determine cytokine recovery. The assays were performed in 96-well filter-plates (Bio-Rad). All incubation steps were performed at room temperature and in dark to protect the beads from light. In brief, the wells were pre-wetted with 100 μl of assay buffer, which was later removed by vacuum suction. The coupled beads (1×) in the kit were vortexed and then transferred (50 μl) to each well. The beads were filter-washed (×2) by using 100 μl wash buffer. The appropriate diluted standards, controls and samples (50 μl each) were then added to each well, and the filter-plates were shaken on a plate-shaker first at 1,100 rpm for 30 s and then at 300 rpm for 30 min. After incubation, the wells were washed (×3) with wash buffer. The beads in the wells were then incubated with detection antibodies (25 μl) on a plate-shaker at 300 rpm for 30 min, and washed (×3) again with 100 μl wash buffer. Fifty μl streptavidin-phytoerythin (1× in assay buffer) was then added to each well, the filter-plates were kept for 10 min, and the wells were washed (×3) with 100 μl wash buffer. Finally, 125 μl assay buffer was added to each well and the plates were shaken at 1,100 rpm for 30 s. The fluorescence intensity of the beads was measured by using a Bio-plex array reader. Bio-plex manager software with five-parametric-curve fitting (Bio-Rad technical note 2861 at http://www.bio-rad.com) was used for data analysis.

### Statistical analysis

The data were analyzed by SigmaPlot Statistical Software 11.0. Multiple groups were analyzed by one-way ANOVA followed by Tukey’s multiple comparison tests, and p < 0.05 was considered significant.

## Results

### Standard curves for recombinant cytokines

**Figure 1:**
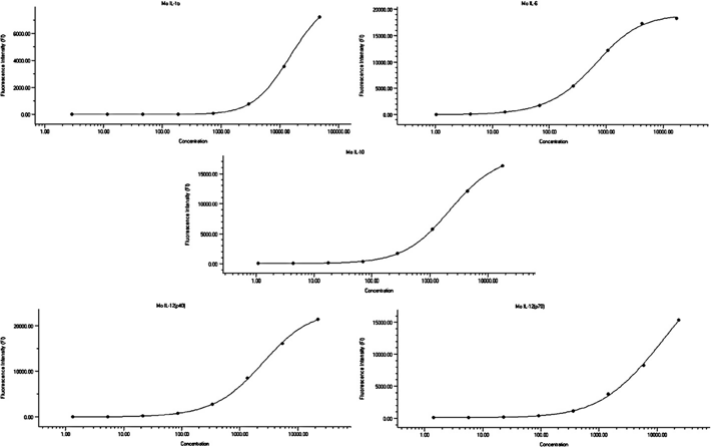
**Standard curves for recombinant cytokines.** Data were generated by combining an eight-fold dilution of the various standards (IL-1β, IL-6, IL-10, IL-12 (p40), IL-12 (p70). The fluorescence intensity (FI) was measured by using the Bio-plex system (Multiplex Suspension Cytokine Array; Bio-Rad, Hercules, CA, USA). Standard curves were quantified and plotted with Bio-Plex Manager software by a five-parameter regression formula.

The Standard curves were generated for recombinant cytokines (IL-1β, IL-6, IL-10, IL-12 p40 and IL-12 p70). The cytokine-specific single beads (5 different bead populations) were identified through sequential gating on doublet discriminator signal and intrinsic bead dye (red *vs* infrared), exclusive of bead aggregates and debris. The concentration of cytokines was measured as mean fluorescence intensity of the streptavidin-PE signal on the surface of the beads, from a minimum of 50 beads/cytokine. The standards were calculated in duplicate for eight data-points. The standard curves were then plotted through a five-parameter logistic curve fitting (Figure 1).

### Cytokine elaboration by live mycobacteria-infected PMs, *in vitro*

**Figure 2:**
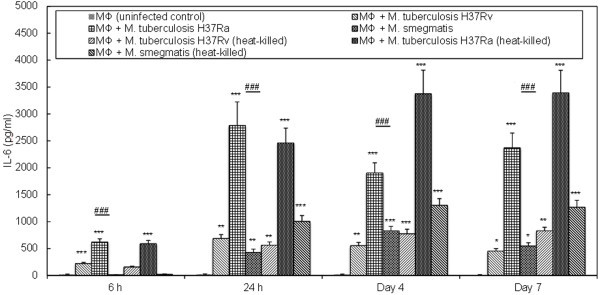
**Determination of IL-6 elaborated by PMs infected with live and dead, and pathogenic (** ***M. tuberculosis*** **H37Rv) and non-pathogenic (** ***M. tuberculosis*** **H37Ra and** ***M. smegmatis*** **) mycobacteria,** ***in vitro*** **.**

The PMs infected with live *M. tuberculosis* H37Ra, as compared to those infected with *M. tuberculosis* H37Rv and *M. smegmatis*, invariably, elaborated significantly (p < 0.001) augmented levels of IL-6 in their culture medium (Figure 2). Kinetically, the increased IL-6 elaboration could be observed as early as at 6 h, which reached its maximum (2785.75 ± 438.05 pg/ml) at 24 h, dipped slightly (1904 ± 72.12 pg/ml) on Day 4, and then almost plateaued thereafter till Day 7 (2372 ± 275.77 pg/ml) observed. On the other hand, IL-6 elaboration by *M. tuberculosis* H37Rv-infected PMs, though significantly (p < 0.001) low as compared to that elaborated by *M. tuberculosis* H37Ra-infected PMs, could also be observed as early as at 6 h, which reached its maximum at 24 h (688.25 ± 33.59 pg/ml; nearly 4-fold less as compared to that elaborated by *M. tuberculosis* H37Ra-infected PMs), and then plateaued thereafter till Day 4 (552.5 ± 19.09 pg/ml) and Day 7 (454.5 ± 16.26 pg/ml) observed. Notably, IL-6 elaboration by the PMs infected with *M. smegmatis*, unlike *M. tuberculosis* H37Ra and *M. tuberculosis* H37Rv, could only be observed as early as at 24 h (427 ± 62.23 pg/ml), reached its maximum on Day 4 (830.25 ± 35 pg/ml) and then returned back to near 24 h levels on Day 7. Curiously, none of these live mycobacteria induced the PMs for any significant increase in the elaboration of IL-1β, IL-10, IL-12 p40 and IL-12 p70 (data not shown).

### Cytokine elaboration by heat-killed mycobacteria-infected PMs, *in vitro*

The PMs infected with both live and heat-killed *M. tuberculosis* H37Ra, separately, at both 6 and 24 h, elaborated IL-6 in quantities that were not significantly different from each other; however, on both Day 4 and Day 7, PMs infected with the heat-killed *M. tuberculosis* H37Ra, as compared to those infected with the live ones, elaborated significantly (p < 0.001) higher levels of IL-6 (3375.25 ± 78.84 pg/ml and 3392.5 ± 62.93 pg/ml on Day 4 and Day 7, respectively; Figure 2). Similarly, PMs infected with both live and heat-killed *M. tuberculosis* H37Rv, separately, elaborated IL-6, at both 6 and 24 h, in quantities which were not significantly different from each other; however, on both D4 and D7, the heat-killed *M. tuberculosis* H37Rv elaborated significantly higher levels of IL-6, as compared to the live ones. Invariably, *M. tuberculosis* H37Ra-infected PMs, as compared to those infected with *M. tuberculosis* H37Rv, irrespective of live or heat-killed bacilli, at all the time-points studied, elaborated IL-6 in quantities that were significantly (p < 0.001) higher. The PMs infected with both live or heat-killed, *M. smegmatis*, in contrast to those infected with *M. tuberculosis* H37Ra and *M. tuberculosis* H37Rv, at 6 h, did not elaborate significantly enhanced levels of IL-6 as compared to that elaborated by the uninfected control PMs; however, at 24 h and on both Day 4 and Day 7, the heat-killed *M. smegmatis*-infected PMs elaborated significantly (p < 0.001) higher levels of IL-6 as compared to those infected with the live ones. As in the case of live mycobacteria, none of these heat-killed mycobacteria, induced the PMs for any significant increase in the elaboration of IL-1β, IL-10, IL-12 p40 and IL-12 p70 (data not shown).

## Discussion

Our laboratory has been, for over last 15 years, engaged in molecular studies related to the host-pathogen interaction during *M. tuberculosis* infection. More pointedly, the identification of biomarkers of mycobacterial infection, consequent to mycobacteria and MΦ interaction, *in vitro*, has been our major focus. In the present studies, our most important and striking observation has been that mycobacteria, irrespective of their viability, virulence and pathogenicity, following their interaction with the PMs, *in vitro*, induced them to elaborate significantly (p < 0.05) enhanced levels of IL-6 only; the enhancement of the elaboration of other studied cytokines (IL-1β, IL-10, IL-12 p40, and IL-12 p70) was not significant. Because a 5-plex cytokine array (IL-1β, IL-6, IL-10, IL-12p40 and IL-12p70) was used, it is possible that other cytokines, if up-regulated, would have missed quantification. However, literature survey has shown that in most of such studies including those using human MΦs, mainly TNF, IFN-γ and GM-CSF are up-regulated, albeit not the extent we have observed enhanced IL-6 elaboration. Nevertheless, future studies using larger plex cytokine arrays may shed more light on this point. Therefore, our results, for the first time, report that IL-6 appears to be the only cytokine whose elaboration is enhanced significantly (p < 0.05) following the interaction of PMs with mycobacteria, *in vitro*.

In the recent past, a great significance has been attached to the identification of biomarkers, also known as biological indicators, of TB. According to Parida and Kaufmann (2010) a biomarker can be defined as a “characteristic that is objectively measured and evaluated as an indicator of a physiological or pathological process or pharmacological response(s) to a therapeutic intervention” (Parida and Kaufmann, 2010). However, so far, no biomarker(s) has been identified that can be used effectively for TB. This probably is one good reason that we still do not have suitable drugs, vaccines and diagnostic tests for TB. Therefore, there is an urgent need to have a suitable biomarker or a group of markers (also called as a biosignature) that is a true statistical reflection and is pathophysiologically related to the clinical outcome of TB, with or without therapeutic intervention. In the present study, apparently for the first time, an effort has been made to project the elaboration of IL-6, as a sequel to the interaction of PMs with mycobacteria, *in vitro*, as a potential biomarker of mycobacterial infection, either stand-alone or along with other cytokines, which may be developed further as a biomarker/biosignature of TB.

The concept of cytokines to function as biomarkers is well established and has been reviewed recently (Doherty et al., 2009), and it has been suggested that both IFN-γ and TNF-α can function as excellent biomarkers for the clinical assessment of TB, as their expression and elaboration has been thought to have important bearing on the immunopathogenesis of TB. Additionally, as both of these cytokines are induced and expressed rapidly early in the infection, they can be detected and quantified much before the onset of the symptoms of TB. Because IFN-γ is induced in a pathogen-specific manner, it thus constitutes a preferred biomarker. IL-4 mRNA expression has been reported to increase in healthy individuals who later develop active TB, and decrease soon after the treatment. Wassie et al. (2008) have reported that in TB patients mRNA expression for IL-4δ2, an IL-4 antagonistic splice variant, appeared to be similar to that of IFN-γ. Rhodes et al. (2007) have reported that there is a strong possibility that the ratio of the levels of IL-4 and IL-4 δ2 may correlate with the severity of TB, and thus may serve as a true reflection of the disease. This contention is further supported by a recent report (Siawaya et al., 2008), which indicates that quick changes in the ratio of IL-4 and IL-4δ2 levels during TB treatment may serve as good indicators of further treatment outcome. Augmented IL-6 elaboration can occur during various infections or inflammation. C-reactive protein, an APP, and other markers of inflammation have also been suggested to function as biomarkers of TB (Doherty et al., 2009). IL-6 is a known inducer of APPs, and Singh and Kaur (2006) have reported a near parallel increase in both serum amyloid P-component (SAP), an APP in mice, and IL-6, in *M. tuberculosis*-infected mice. Our results which demonstrate the elaboration of only IL-6 as the major cytokine following the interaction of PMs with mycobacteria, *in vitro*, also seem to be in consonance with earlier reports (Mattos et al., 2010; El-Ahmady et al., 1997). Therefore, IL-6 can be expected to be developed as a potential biomarker of TB, either stand-alone or along with other cytokines.

The mechanisms by which *M. tuberculosis* and *M. smegmatis* induce MΦs to elaborate of IL-6 are not yet properly understood. However, the lipoarabinomannan (LAM) present in the *M. tuberculosis* cell wall, independent of lipopolysaccharide, in a dose-dependent manner, has been reported to induce MΦs to elaborate IL-6, *in vitro* (Zhang et al., 1994). The LAM-induced mycobacterial response elements (transcription factors) NF-IL6 and NF-kB on the IL-6 gene, following their binding with the IL-6 regulatory region, regulate the expression of IL-6 gene (Zhang et al., 1994). A similar molecular mechanism(s) of IL-6 induction may also be operational in our studies reported herein.

We have observed that of all the cytokines studied, invariably, only IL-6 elaboration was enhanced significantly (p < 0.05). Though, apparently, there is no explanation for these observations, the following reports appear supportive. Denis (1992) has demonstrated that in tissue culture medium, which does not support full growth, both human and mouse rIL-6 function as potent growth factors for four virulent strains of *M. avium*; IL-6 was rapidly taken-up by the mycobacteria through a single receptor species of 50 nM Kd with approximately 15, 000 receptors/mycobacterium. It may just be possible that mycobacteria, including *M. tuberculosis*, may induce the augmented elaboration of IL-6 to support their own growth. Presently, our lab is engaged in the generation of more specific evidences to this effect.

The PMs infected with live *M. tuberculosis* H37Ra, as compared to those infected with *M. tuberculosis* H37Rv and *M. smegmatis*, invariably, elaborated significantly (p < 0.001) enhanced levels of IL-6. A major difference exists between phagosomes containing pathogenic and non-pathogenic mycobacteria. Phosphatidylinositol 3-phosphate (PI3P), a membrane trafficking regulatory lipid, is essential for the phagosomal acquisition of lysosomal contents (Vergne et al., 2005). *M. tuberculosis* H37Rv secrets a lipid phosphatase called SapM, which hydrolyzes PI3P and thus inhibits the fusion of phagosomes with late endosomes (Saleh and Belisle, 2000). Non-pathogenic mycobacteria, on the other hand, do not secrete SapM, and thus no inhibition of the fusion of phagosomes with late endosomes occurs. Because phagosome-lysosme fusion is known to result in enhanced elaboration of cytokines, it may be the only possible reason or one of the possible reasons for the augmented elaboration of cytokines by non-pathogenic mycobacteria. Another reason for the differential elaboration of cytokines by pathogenic and non-pathogenic mycobacteria may be because of the reported difference in their LAM structure. LAM from non-pathogenic mycobacteria is characterized by its extensive arabinose side-chains, and is an extremely potent inducer of cytokines; paradoxically, LAM from pathogenic mycobacterial species, in which short mannan segments extensively mask the arabinan side chain, is approximately 100-fold less potent (Chatterjee et al., 1992). The differences in the LAM structure have also been associated with the ability of mycobacteria to survive and replicate within MΦs, and to cause a productive infection. It is thus possible that *M. tuberculosis* H37Ra and *M. smegmatis* may not be able to cause productive infections because of the continuous triggering of the release of cytokines by the MΦs infected with them. Thus, these infected MΦs acquire bacteriostatic activity and possibly, other phagocytic cells may accumulate around them, leading to granuloma formation. The inherent cytotoxicity of *M. tuberculosis* H37Rv for MΦs may also be responsible for the diminished elaboration of cytokines (Falcone et al., 1994). As various *M. tuberculosis* field isolates are known to differ in their virulence and pathogenicity, in the present studies we considered it expedient to include both avirulent and virulent, and non-pathogenic and pathogenic mycobacteria.

The heat-killed mycobacteria, as compared to their live counterparts, induced the infected PMs for enhanced elaboration of IL-6. The live mycobacteria are known to inhibit phagosome-lysosome fusion, whereas heat-killed ones do not (Barker et al., 1997), possibly because of the enhanced secretion of sulphatides by live mycobacteria as compared to the heat-killed bacilli (Gordon et al., 1980). Further, PI3P is known to be retained on the phagosomes which contain heat-killed mycobacteria, but is continuously eliminated from those having live bacilli (Vergne et al., 2005). The ability of the heat-killed mycobacteria to induce more cytokines may also partially reflect the loss of direct cytotoxic effect produced by the viable mycobacteria on infected MΦs (Falcone et al., 1994). Higher IL-6 production by heat-killed mycobacteria may simply reflect that once killed, these mycobacteria may lose partly or completely some of their heat-labile virulence components, and thus may allow greater phagosome-lysosme fusion in infected MΦs.

The human relevance of these studies remains an important point to ponder. Apparently, there is no specific experimental or clinical evidence(s) to suggest the extrapolation of the results of cytokine elaboration, especially IL-6, following murine MΦ and mycobacteria interaction, *in vitro*, to human situation. However, there are several reports of the interaction of human MΦs and mycobacteria (Beltan et al., 2000; Blanchard et al., 1991), which show results similar to those reported herein. Therefore, it seems plausible to objectively extrapolate the results of this study to humans both for clinical and latent TB, albeit after expedient extended studies, both in murine and human systems. Nevertheless, the extrapolation of results generated *in vitro* using an isolated population of cells may just be a gross oversimplification of the events occurring *in vivo*, and thus may require a more complex culture system or a suitable animal model, which should mimic situation in human TB patients, as closely as possible. Additionally, appropriate translational researches are warranted to make these studies applicable to humans.

In conclusion, our results demonstrate that the interaction of PMs with mycobacteria, *in vitro*, invariably, ensued in the augmented elaboration of IL-6 only, and thus, it may be used as a potential biomarker (stand-alone) or as a biosignature (along with other cytokines), of mycobacterial infection. Such a biomarker/biosignature is warranted to aid in the detection of mycobacterial infection, and also, possibly, to give a fillip to the faster progression of the potential anti-TB drugs presently stagnating in the clinical trial pipe-line, albeit after due caution and skepticism.
